# Introductory Address before the Class in the Ohio Dental College

**Published:** 1874-12

**Authors:** Wm. Clendenin


### DENTAL REGISTER.

Vol. XXVIII.] DECEMBER, 1874. [No. 12.

### INTRODUCTORY ADDRESS BEFORE THE CLASS IN THE OHIO DENTAL COLLEGE, Oct. 17, 1874.

BY PROF. WM. CLENDENIN, M. D.

The study of anatomy, undertaken in the right spirit, is one of the most interesting and useful of studies. The human form is nature's master-piece, and its contemplation is one of the noblest exercises of the mind; it induces us to look a little beyond "final purposes," and tends to give additional force to the conviction derived from more direct revelation of the infinite wisdom, power and goodness of the Creator. The human form is instructive because related to the intelligent will of man on the one hand, and to the various members of the animal kingdom on the other, and hence it engages the attention of the philosopher and the naturalist. The human form has ever been the favorite subject of the painter and the sculptor. No one can doubt that the unquestionable influence of the great masters (in art), was owing to a knowledge of anatomy. Without this science the truth in art could never have been discovered. And when we see the exaggerations of artists, it is not science which is at fault. It is the misapplication of science which has injured art. Certain deep muscles exert the highest influence over the external form, yet these should not be exaggerated as has been done by some artists, and even by Michael Angelo himself. The visitor to that temple of taste—the Tribune of the Florence gallery will be convinced that the author of that great statue that enchants the world—the uninimitated, inimitable Venus, was an anatomist.

Anatomy is the basis of many sciences, but it is to the physician and surgeon that a knowledge of anatomy is especially important and absolutely necessary. The knowledge of the physician regarding the nature of disease and disease processes has advanced simultaneously with that of general anatomy. Bichat who first separated the body into its elementary parts, thus became the founder of modern special pathology. And we have since learned how general anatomy, reduced from physical properties of parts and crude observations, may coincide with some minute investigations of a chemical and microscopical kind. Still later Culler and Hunter observed how the application of distinction of tissues was made to illustrate the nature of disease-action. And yet while it was reserved for Bichat to complete a more perfect system of general anatomy, we must not forget that Smith in 1790, applied his knowledge of external anatomy to elucidate the nature of disease. And Pinel, after him, made the distinction between the membranous and other animal structures the foundation of his pathology. Sylvius and Bœhaava, in their day supposed that by various hypothetical processes, all of a chemical nature, the blood became a collection of various juices, and diseases were accounted for by a supposed prevalence of one or the other acrid principles—the acid or the alkaline. The splendid results obtained by Galileo and Newton led to the application of mechanical principles to the explanation of those phenomena which we call disease. And for many years the minds of physicians were occupied in vain endeavors to account for, and explain the phenomena of life, on mechanical principles. The main points to which the mechanical school attended were, the application of mechanical calculations to the force of muscles, and of hydraulic reasonings to the motion of the fluids of the animal system; that all the properties of bodies and their mutual operations might be accounted for by supposing them constructed of particles of various forms, round or angular, pointed or hooked, straight or spiral. Lucretius undertook to explain why wine passes rapidly through a sieve and oil slowly, by telling us that one has its particles larger than the other, or more hooked and interwoven together; that the secretions of bile, pancreatic juice, tears, milk etc., were affected by the organs operating after the manner of sieves. Thus while one of these sects (chemists and mechanical) had neglected the effect of the solids of the living frame; the other attended only to these. And even these were considered only as canals, as cords, as levers, as lifeless machines. These reasoners never looked for any powers of a higher order than the cohesion, the resistance, the gravity, the attraction which operated in inert matter. If the chemical school assimilated the physician to a vinter or brewer; the mechanical physiologist made him a hydraulic engineer, and it was not until the anatomist Harvey taught us how the blood circulated between the heart and the lungs, and the remote parts of thebody, that these absurd notions were appreciated and corrected.

But while we admit the great importance of anatomy to the physiologist and pathologist, to the physician and surgeon, a more pertinent inquiry, for you, gentlemen, is what practical advantage is a knowledge of anatomy to the dental surgeon? In answering this question, we have only to remember that dentistry includes the treatment and care of those parts and organs which are immediately concerned in the preparation and digestion of food, and, therefore, of the nutrition and repair of the natural waste of all the tissues of the body. Consider for one moment the wonderful mechanism, and the important functional associations of the teeth, the jaws, the mouth, with their vast and complicated nervous and vascular system, and we are prepared to admit the statement made by our ablest writers, viz: that the irritation caused by the deranged or morbid eruption of the teeth, is a prime cause of almost every disease of childhood. The form, position, relation and associations of these most important organs, constitutes an essential and intricate portion of the anatomy of the human body. But to understand the mutual relations and sympathies between the teeth and the other organs of digestion, as well as more distant but not less intimately associated organs, a knowledge of the whole body is absolutely necessary. The ears, the eyes, the nose, the mouth, the palate, tongue, cheek, scalp, salivary glands, muscles of the neck are all directly associated with the teeth through the branches of the fifth pair of nerves; and through each of these, other nervous and vascular associations and sympathies are formed more or less directly. It must, therefore, be apparent that we can not understand, so as to be able to treat intelligently, the diseases of any particular portion of the body without a knowledge of these organs and parts with which it is anatomically connected.

The functional association of parts is a subject which has not, as yet, received the attention its importance demands. The associations here referred to, is that which is accomplished through the nervous system, a careful study of which is required to enable us to read aright the various phenomena of diseases. An inflamed joint is flexed and fixed, while the temperature of the surrounding skin is sensibly increased above the normal temperature. The fact that the same nerve trunk which supplies the interior of a joint; supplies the muscles which move the joint, and also the skin which covers the muscles points directly to the manner which the symptoms are produced. The soft parts, including the integument, are thus brought into functional association with the interior of the joint, the seat of the inflammation. Many of the muscles of different parts of the body have various functional associations, derived from the different nerves distributed to them. The omo-hyoideus muscle receives its nervous supply from three different sources, and is thus associated in its functions with the muscles of the neck, the muscles concerned in deglutition and those of respiration. Examples of this kind are very numerous, but enough has been said to show the student of dental medicine the necessity of a knowledge of the anatomy of the whole body, as well as of the teeth, jaws, mouth and palate. The spirit and preparation we bring to the study of any science is a matter of no little consequence. If we would study with profit a work of literature, we would first endeavor to make ourselves acquainted with the genius of the author, also his previous labors, and the circumstances under which his work was executed. Without this, although we may perhaps enjoy its perfection as a whole, and admire the beauty of its detail, yet the spirit which pervades it will escape us, and many passages may forever remain unintelligible.

So in the study of nature, we may be astonished at the infinite variety of her products; we may even study some portions of her work with enthusiasm, and nevertheless remain strangers to the spirit of the whole; ignorant of the plan on which it is based, and fail to acquire a proper conception of the varied affinities, which combine beings together so as to make of them that vast picture in which each animal, each plant, each group, each class has its place, and from which nothing could be removed without destroying the proper meaning of the whole.

In the study of anatomy the student too often loses sight of the fact that the human body is to be examined in respect to its own organism, and also in its relations to creation as a whole; because he who beholds in nature nothing besides organs and their functions, may persuade himself that the body is merely a combination of chemical and mechanical actions, and he is therefore in danger of becoming a materialist. While on the other hand, he who considers only the manifestations of intelligence and creative will, without considering the means by which they are executed, and those laws by virtue of which all beings preserve their characteristics, will be liable to confound the Creator with the creature. Matter and mind must be contemplated at the same time, so that we can see in creation the execution of a plan fully matured in the beginning, and undeviatingly pursued—all created beings, (nature), regulated according to the immutable laws of God.

In the primitive ages of the world, anatomy was little cultivated as a science, and hence the art of surgery was undeveloped. Religious scruples forbade the opening of the human body to inspect the organs contained within it; and it was only by dissecting the lower animals that a knowledge of the internal organs was obtained, which might serve as a guide to understand similar organs and functions in the human body. Aristotle was the first to give accurate descriptions of the internal organs of different species of animals, and for many centuries after him, little was done to advance the science of anatomy by actual dissection. The first step taken toward the cultivation of anatomy as a science was at Alexandria, in Egypt, during the reign of the Ptolemies, 300 B. C. Galen mentions Erosistratus of Chios, and Herophilus of Chalceden as eminent anatomists of the school of Alexandria. The bodies of criminals were opened by permission. But the greatest steps in advance were taken by Galen, the great physician of Pergamos, who was born at Alexandria, A. D. 131. He showed that the arteries contained blood and not air as had formerly been supposed by his predecessors. During the middle ages anatomy was neglected together with the natural sciences, or cultivated only by the Arabs. During the fourteenth century the spirit of religious liberty revived the cultivation of the arts and sciences in Italy; and Luzzi, professor of anatomy at Bologna, first publicly dissected two human bodies in the presence of medical students, and soon after published a description of the organs from direct observation and dissection. The works of Luzzi and Galen served as text books on anatomy until the sixteenth century, from this period onward anatomy was studied from actual dissection in Italy —hence it is that we find many organs still bear the names of those eminent Italian physicians who first dissected the human body.

But popular prejudice has always, and still hinders the free dissection of human bodies, except in some parts of Europe. Yet the love of scienee is sufficient in most civilized nations to permit the study of anatomy, at least to an extent necessary for its development as a basis of correct knowledge of the human body.

Anatomy is now justly regarded as one of the most important branches of natural science, and it is at present divided into various departments, viz: human anatomy, comparative anatomy, and these subdivided into distinct branches, under the names of surgical anatomy, descriptive anatomy, histological anatomy, general anatomy, and microscopic anatomy. In prosecuting the study of either of these departments of anatomy, models, manikins, charts, and diagrams are useful aids, but it is only by dissecting the human body that a proper idea can be formed of the appearance, the form, the position and relation of the several parts or organs. Again, every art and science has a language of technical terms peculiar to itself. With these terms every student must make himself familiarly acquainted at the outset. Anatomy has its technical terms, without a knowledge of which the student will meet with difficulties and embarrassments at every step—indeed, some knowledge of the Latin and Greek languages is necessary to a perfect understanding of anatomical terms, No student should allow himself to pass over a word or phrase without fully informing himself of its meaning,

